# Genistein: Dual Role in Women’s Health

**DOI:** 10.3390/nu13093048

**Published:** 2021-08-30

**Authors:** Linda Yu, Eddy Rios, Lysandra Castro, Jingli Liu, Yitang Yan, Darlene Dixon

**Affiliations:** Molecular Pathogenesis Group, Mechanistic Toxicology Branch (MTB), Division of the National Toxicology Program (DNTP), National Institute of Environmental Health Sciences (NIEHS), National Institutes of Health (NIH), Research Triangle Park, Durham, NC 27709, USA; yu1@niehs.nih.gov (L.Y.); eddy.rios@nih.gov (E.R.); castro@niehs.nih.gov (L.C.); jingli.liu@nih.gov (J.L.); yitang.yan@nih.gov (Y.Y.)

**Keywords:** genistein, soya products, dual role, dose-dependent

## Abstract

Advanced research in recent years has revealed the important role of nutrients in the protection of women’s health and in the prevention of women’s diseases. Genistein is a phytoestrogen that belongs to a class of compounds known as isoflavones, which structurally resemble endogenous estrogen. Genistein is most often consumed by humans via soybeans or soya products and is, as an auxiliary medicinal, used to treat women’s diseases. In this review, we focused on analyzing the geographic distribution of soybean and soya product consumption, global serum concentrations of genistein, and its metabolism and bioactivity. We also explored genistein’s dual effects in women’s health through gathering, evaluating, and summarizing evidence from current in vivo and in vitro studies, clinical observations, and epidemiological surveys. The dose-dependent effects of genistein, especially when considering its metabolites and factors that vary by individuals, indicate that consumption of genistein may contribute to beneficial effects in women’s health and disease prevention and treatment. However, consumption and exposure levels are nuanced because adverse effects have been observed at lower concentrations in in vitro models. Therefore, this points to the duplicity of genistein as a possible therapeutic agent in some instances and as an endocrine disruptor in others.

## 1. Introduction

### 1.1. Genistein in Food

Genistein (5,7-dihydroxy-3-(4-hydroxyphenyl) chromen-4-one) is a phytoestrogen and isoflavone found in soybeans and soy-derived foods [1], including soya products, meat alternatives, edamame, and tempeh [2]. It has been detected in many processed foods [3] and can also be found in other foods [4]. Genistein’s content in mature soybean seeds and therefore in soya products, varies by region, from the highest genistein content (>70 mg/100 g food) in soybean seeds from the US, Korea, and Japan, to the lowest from Europe (39.78 mg/g) and Taiwan (45.88 mg/g) [4]. Alongside reporting high genistein concentrations in soybeans, the US is currently leading the world in soybean production and export [5]. Furthermore, the demand for plant-based protein in Western societies is increasing; while dollar sales of all US foods have increased by 17% over the past two years, plant-based food sales have increased by 43% over this same time period [6]. As a compound that is so commonly found in food and specifically in soy products (Table 1), developing our understanding of genistein is paramount in both preserving global health and furthering advancements in women health.

### 1.2. Genistein Levels in Various Populations

Genistein has been found and quantified globally in measures ranging from daily intake to serum concentration. Prominent data from several different studies in populations are outlined in Table 2, which is representative of two larger correlations of serum genistein concentrations.

First, Table 2 shows that individuals who consume more soy products or soy-derived foods have higher serum levels of genistein. Supporting data from Verkasalo et al. [63] demonstrated that among four groups of twenty British women consuming increasing amounts of soya products (determined via a food diary method), plasma concentrations of genistein increased in a manner correlated with total soya consumption. In order of lowest to highest soya product consumption, the participant groups’ geometric mean plasma concentrations (nmol/L) of genistein were 14.3, 16.5, 119, and 378. The Spearman correlation coefficient a quantitative measure for the strength of this correlation between plasma isoflavone concentrations and estimated dietary intakes was determined to be between 0.66 and 0.80 [63]. This correlation also extends beyond blood serum, as genistein has been shown to be more common in the breast milk of mothers that are consuming vegetarian and especially vegan diets [3]. It was further demonstrated that genistein can cross the placental barrier and potentially affect the developing fetus, as it was detected in similar concentrations in the maternal plasma, umbilical cord plasma, amniotic fluid, and neonate plasma in seven healthy Japanese mother-child pairs [64].

**Figure 1:**
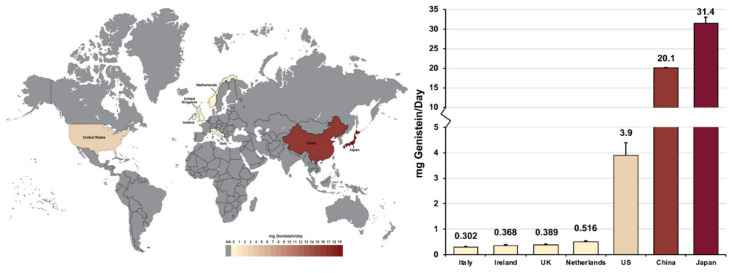
Heatmap showing daily genistein consumption levels across 7 surveyed countries. Data from [58,59,61,62].

Secondly, the data in Table 2 also shows that both residents of Asian countries and Asian minority populations in Western countries consume significantly more soy products and, therefore, have higher serum genistein levels than other populations. This is further displayed in Figure 1, and validated when considering that China is the world’s largest importer of soybeans, consuming roughly one-third of the global annual soybean harvest [65]. However, evidence suggests that eating a soya-rich diet, as vegetarians and vegans commonly do [66], can elevate daily genistein intake levels among individuals to a similar degree. British women who consumed soya regularly were reported to have a daily soya-product consumption that rivals that of Japanese adults consuming a traditional diet [63].

Another relevant population in relation to genistein is perimenopausal and post-menopausal aged women. As literature regarding the clinical applications of phytoestrogens has risen to prominence, millions of peri- and postmenopausal women have begun taking genistein and soya supplements, aiming to alleviate their menopausal symptoms [67,68]. Taken in conjunction with the aforementioned populations, it is important to investigate genistein’s effects, not only because of increased global soya consumption [69], but because genistein is ubiquitously present in food, breast milk, and human serum and is therefore bioactive in vulnerable, underrepresented, maternal, and neonatal populations.

### 1.3. Metabolism and Metabolites of Genistein

Genistein is typically ingested from vegetation as the glycoside genistin. Genistin is hydrolyzed by phlorizin hydrolase (a small intestine brush-border lactase) [70] or by enteric microflora [71] into genistein (the bioactive aglycone) before absorption or further modification by enteric microflora [72]. Genistein, like other polyphenols, has an oral bioavailability of roughly 10% [73]. With its low absorption potential, its lipophilic nature, and low molecular weight, genistein can be passively transported into intestinal cells [74], leading to post-absorption metabolism.

Most orally consumed genistein is eliminated by urine within a day of consumption [75]. When absorbed into the bloodstream via the intestinal tract, genistein and all of its metabolites were shown in a mouse model to have a half-life of 46 h [76]. However, in a study of nineteen healthy women, its bioactive life as unconjugated genistein aglycone was shown to be much shorter at just 7.13 h [77] This short bioactive life most commonly ends when genistein is modified by uridine diphosphate-glucuronosyltransferases (UGTs) and sulfotransferases (SULTs) in the intestinal enterocytes and liver [73,78]. Conversion to genistein glucuronide is the most common fate of absorbed genistein [76,79], and though it varies greatly between individuals, it is also less commonly carried out by UGTs in the kidneys [80]. The large majority of the circulating genistein that is not converted to a glucuronide form is converted via enterocytic and hepatic sulfotransferases (SULTs) to a sulfate form [76]. These sulfate and glucuronide groups are added to the 7 and 4′ positions, creating different compounds that can have 1 glucuronide, 1 sulfate, 2 glucuronides, or one of each [79]. It is also important to mention that sufficient expression and localization of UGTs and SULTs in other organs, such as the heart and lungs, allows for minor metabolism of genistein in these organs [78,81]. Furthermore, different cell types, due to the composition of different ratios of UGT:SULT enzymes, may vary in their metabolism of genistein [82].

**Figure 2:**
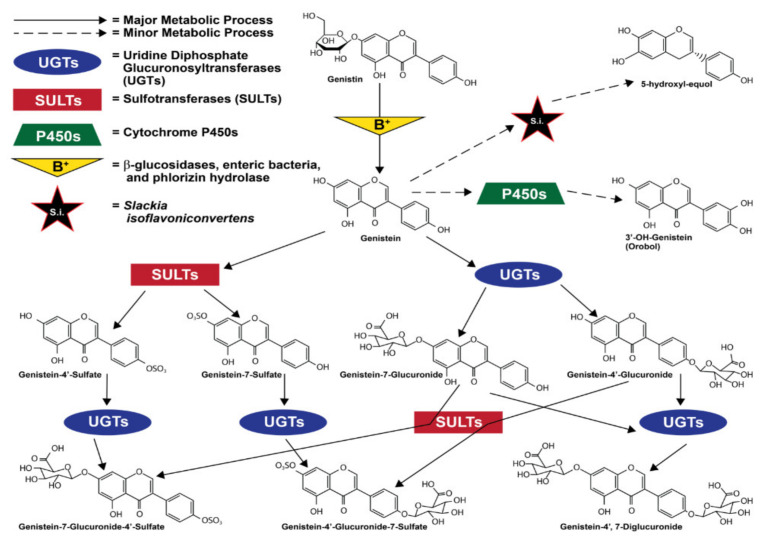
Major genistein metabolites. Data from [79,82,86,87,88,89]. Structures from [1,79].

To a significantly lesser extent [83,84], genistein is also metabolized via cytochrome P450 (CYP) reaction to produce mostly hydroxylated metabolites [84,85,86]. The enzyme CYP1A2 is the most relevant of the CYP group, converting genistein to orobol (3′-OH-genistein) [86,87]. Less often, other CYP enzymes such as CYP2E1, CYP2D6, and CYP3A4, and CYP2C8 also metabolize genistein via oxidation [86,87]. Figure 2 illustrates ingested genistein’s most common metabolic processes and metabolites.

Though enteric bacteria are known to play a prominent role in the uptake and metabolism of genistein [3], Munro et al. [90] reported that its metabolic pathway may be significantly altered by variations in microflora, intestinal transit time, pH, redox potential, and even immune status and diet. There is even a likely temporal aspect to this metabolic plasticity, as data presented in Hoey et al. [91] suggests that the ability to hydrolyze glycosides to aglycones, and therefore genistin to genistein, develops before 4–6 months postnatally and plays an important role in isoflavone metabolic capabilities.

Equol, metabolized from daidzein, another isoflavone found in soybeans, is also relevant when discussing enteric bacteria and genistein because it is an isoflavone metabolite with stronger estrogenic activity than all other known isoflavones and isoflavone metabolites; it also exhibits the strongest antioxidant activity of any isoflavone metabolite [92,93,94]. Although equol itself is produced when intestinal bacteria metabolize daidzein and its glycoside form daidzin, the human microfloral bacteria *Slackia isoflavoniconvertens* has also been described as capable of converting genistein to 5-hydroxy-equol [88]. However, while this metabolite is slightly altered, 5-hydroxy-equol shares many of equol’s chemical properties, exhibiting a greater antioxidant capacity than genistein [95].

It is difficult to quantify the significance of this genistein metabolite in terms of women’s health, as most women likely do not have the correct gut bacteria for producing it. Depending on genetic and dietary factors, only 25–50% of people are believed to have gut bacteria that are capable of producing equol from daidzein [96]; this is especially relevant considering multiple different bacteria can catalyze this conversion, and only a fraction of them are known to be concurrently capable of 5-hydroxy-equol formation from genistein.

### 1.4. Estrogenic Effects of Genistein

Given the structural similarity of genistein and estrogen, genistein may exhibit a litany of possible biological effects while circulating. Many of these effects stem from its status as an isoflavone and therefore an estrogen mimic [75]; it acts on estrogen receptors (ERs), ER alpha and beta, primarily through the classical genomic mechanism [97]. It does differ from estrogen, however, in its preference for ER beta (gene name: ESR2) over ER alpha (gene name: ESR1). In a solid-phase competition experiment, genistein was shown to have a binding affinity for ER alpha that is 4% of that of 17 β-estradiol (E2), and a relative binding affinity for ER beta of 87% [97]. Because of genistein’s hydroxyl substituents, these relative binding affinities for both ERs are significantly higher than that of other isoflavones, such as daidzein and formononetin [97]. This, however, is complicated by variation in the presence and distribution of both ERs temporally, between different body tissues and cell types, and even between individuals and populations [98,99,100].

Genistein has also been shown to exhibit agonistic activity with G protein-coupled estrogen receptor 1 (GPER1) [101], yielding a binding affinity higher than that of E2 but smaller than that of E2 [101,102]. This activity is compounded by results reported in Du et al. [103], in which treatment with genistein induced greater gene and protein expression of GPER while inhibiting MAP kinase activation in mouse microglial cells. Other molecular targets of genistein include topoisomerase I and II [104,105], protein tyrosine kinases [106], and 5α-reductase [107].

## 2. Biological Effects and Mechanism(s) of Genistein

### 2.1. In Vivo Experimental and Clinical Findings

Before soy gained widespread usage and more media attention, genistein was thought to be a primarily beneficial chemoprotective compound in vivo. Barnes [108] created a table detailing 29 studies characterizing the effects of genistein and genistein-containing products on carcinogenesis in rats and mice, finding a protective effect of genistein in 21 studies, and no effect in the other 8 studies. In vivo evidence also supports genistein’s capability for supporting bone health and suppressing cancer development in tissue. Messing et al. [109] treated pre-operative bladder cancer patients with daily oral genistein (placebo, 300, or 600 mg per day), finding that once excised, the cancerous bladder tissue had significantly lower levels of EGFR phosphorylation. Among its reported antitumor, osteoblastic, and anticarcinogenic abilities, genistein has also been suggested to exhibit antioxidant [110], positive cardiovascular [111], and antilipogenic [112] effects. Although many subsequent reviews have echoed these positive findings, there exists some recent controversy over genistein’s net beneficial effects.

Given its strong potential for therapeutic activity, genistein has faced more scrutiny over the past decade; it does have the potential to exhibit adverse effects. Turner et al. [113] found that serum genistein levels that correlate with those found in women consuming a high-soy diet did not affect bone loss in a rat model for postmenopausal osteoporosis; this evidence outright contradicts previous literature on genistein’s osteoblastic capabilities [114,115].

Singh et al. [116] demonstrated that a single high dose of genistein (500 and 1000 mg/kg) had hepatotoxic, oxidative stress, and correlative genetic expression effects within 24 h of intraperitoneal administration into male Swiss albino mice. However, this was not the case with lower doses; thus, these negative effects were elicited by levels of circulating genistein that was within the realm of pharmacological treatment [109].

Studies with rats have also demonstrated that peri- and neonatal exposure of rats to genistein can negatively affect their reproductive capabilities. Wisniewski et al. [117] found that exposing male rats to even low doses of genistein during gestation resulted in significantly decreased phallic length, testis size, circulating testosterone, and general reproductive fitness. This is especially concerning considering genistein’s ability to cross the human placental barrier [64]. Lewis et al. [118] also demonstrated that near-therapeutic doses of genistein (40 mg/kg subcutaneously) given to neonatal female rats could cause increased uterine weight, advanced onset of puberty, and even permanent estrus. However, these effects were not replicated in females dosed with 4 mg/kg, correlating with the exposure level for human infants drinking soy-based formula [57].

In vivo studies of genistein’s effects may be further confounded by the timing and frequency of genistein consumption. Kerrie et al. [119] hypothesized that lifetime soy consumption, if begun early in life, causes epigenetic changes that reduce the occurrence and reoccurrence of breast cancer. Some of the most prominent data supporting this claim come from the work of Korde et al. [120], which found the most consistent reduction in breast cancer risk among Asian American women who had their largest soy intakes during childhood as opposed to adolescence or adulthood (although a decrease in risk was seen across all 3 groups). Kerrie et al. further reported that three other case-control studies on Asian and Asian-American women supported the association between reduction of breast cancer incidence and initiation of soy consumption at an earlier age. Interestingly, Joanne et al. [121] reported that although Caucasian women see the same significant benefits, they show less of a reduction in breast cancer risk. However, the idea that genistein’s positive effects are somehow race- or ethnicity-dependent has been mostly discredited, as Asian immigrants to western countries who reduce their soy-intake have a similar cancer incidence as Western individuals [122,123,124].

### 2.2. In Vitro Experimental Findings

Genistein is of particular interest in vitro because it exhibits highly variable and often contrasting biological effects, especially in relation to cell proliferation and cancer. Akiyama et al. [125] showed that, in vitro, genistein inhibited tyrosine-specific protein kinase activity of the EGF receptor, pp60^v-src^ and pp110^gag-fes^, and therefore inhibited growth and metastasis, in A-431 epidermoid carcinoma cells. In vivo, genistein was also shown to inhibit serine- and threonine-specific protein kinase activity in the EGF receptor of these cells [125]. Agarwal et al. [126] reported that, in DU145 metastatic prostate carcinoma cells, genistein inhibited the activation of extracellular signal-related protein kinase (ERK) 1/2, a kinase whose overactivation is a fundamental aspect of prostate cancer proliferation. Treatment of the DU145 cells, a prostate cancer cell line, at doses of 100–200 mM of genistein coincidentally resulted in significant cell growth inhibition and induction of apoptosis [126]. In contrast to this inhibition, Chen et al. [127] demonstrated that lower concentrations (1 mM) of genistein increased the proliferation of MCF-7 human breast cancer cells by increasing the protein and mRNA content of the IGF-1 receptor (IGF-IR) and insulin receptor substrate-1 (IRS-1), enhancing tyrosine phosphorylation of IGF-IR and IRS-1.

**Figure 3:**
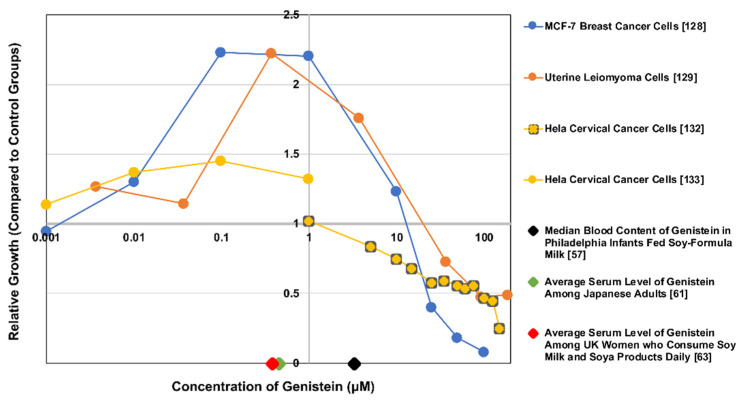
Relative growth of several cell lines at variable concentrations of genistein overlaid with serum levels of genistein for three populations. Data from [57,61,63,128,129,132,133]. Note that no (0) genistein marks a value of 1 relative growth unit. This is considered the control group for all studies. Also, note that none of the cell lines were reported to be prominent metabolizers of genistein. Concurrently, the serum concentration studies only assessed circulating genistein, while its metabolites (likely the most predominant forms in vivo) would presumably also have effects on the cell lines.

However, there is a large body of evidence suggesting that genistein’s metabolism and biological effects may vary by dosage or exposure levels, even depending on the cell type affected. For example, Chen et al. [127] and Wang et al. [128] showed that MCF-7 breast cancer cell growth was stimulated by low concentrations of genistein (10^−8–^10^−6^ M) and inhibited by higher concentrations (>10^−5^ M). Moore et al. [129] found that low in vitro concentrations of genistein (≤1 μg/mL; 3.7 μM) elicited proliferation in human uterine leiomyoma cells, while higher exposure levels (≥10 μg/mL; 37 μM) had inhibitory effects. This non-monotonic dose response to genistein was different for the uterine smooth muscle cells, and a similar dose response to genistein on behavioral parameters in rat offspring has been observed in vivo [130]; it is also a hallmark of environmental endocrine disruptors such as BPA [131]. Figure 3 illustrates this point by portraying three different cell lines’ non-monotonic responses to genistein between 0.001 and 100+ μM. For reference, the dose response curves on this figure are overlaid with serum genistein levels for three surveyed populations.

However, it is important to note that these in vitro studies and reference values do not account for genistein’s metabolites. Given variable bioactivity between genistein and its metabolites, the half-life of genistein and the levels of circulating metabolites may be another pertinent variable in determining the effects of genistein on various tissues in vivo. As such, in vitro studies that treat cells with genistein alone may find significantly different results when compared to studies that used characteristic concentrations of genistein and its metabolites. Further in vitro research should consider genistein metabolites when conducting all forms of exposure assessment, as doing so will provide a more accurate picture of genistein’s net effects.

## 3. Genistein and Women’s Diseases

### 3.1. Genistein and Obesity

Multiple studies purport genistein’s ability to combat obesity at various system-wide levels. This is one of the reasons post-menopausal women may supplement their diet with genistein, as studies with ovariectomized mice have shown orally-consumed genistein to be an inhibitor of the increased fat accumulation, weight gain, insulin resistance, and hepatic lipogenesis that is typically associated with post-menopausal estrogen deficiency [134]. Part of this effect was even shown to be a result of genistein inducing apoptosis in inguinal fat [135].

Genistein was further shown to decrease the adipose tissue content of female mice when compared to vehicle treatment groups [136]. However, genistein’s mechanism of action for reducing body fat likely differs from its activity in other body tissues. While genistein has a much higher binding affinity for ER beta, this same study found that genistein was incapable of significantly reducing the adipose tissue content of female ER alpha knockout mice (αERKO) when compared to a vehicle control [136]. This suggests genistein’s mechanism of action on adipocytes uncharacteristically favors ER alpha rather than ER beta [136], as it did not reproduce this adipose tissue reduction in mice that were lacking ER alpha while expressing ER beta. Further evidence showed that in utero exposures to genistein also potentiates obesity throughout the lives of Agouti mice born to dams consuming high-soy diets during gestation [137], suggesting an expecting dam’s genistein intake may play a role in their offspring’s predisposition towards obesity. Genistein may also regulate obesity by affecting thyroid peroxidase [138], insulin [139], and leptin activity [140].

These anti-obesity effects are not exclusive to the genistein aglycone. For example, orobol (3′-OH-Genistein), the metabolite from the CYP1A2 enzymatic pathway, has been shown to have a significantly greater inhibitory effect on mouse fibroblast adipogenesis than genistein [141].

It is important to mention that genistein’s status as a soy-derived compound means it is generally consumed alongside significant amounts of plant-based protein, and it is well documented that protein is among the most thermogenic and appetite-reducing of the macronutrient groups [142]. Velasquez and Bhathena [143] compiled six human studies, all of which showed that soy-protein was significantly more effective than carbohydrates at increasing metabolic rate and lowering body weight. Though one of these studies did show pork to be more thermogenic than soy [144], three of them showed soy protein to be as or more thermogenic than meat and milk-based proteins [145,146,147]. This means that supplementing one’s diet with genistein from natural sources, and therefore consuming plant-based protein, likely yields many of the thermogenic benefits associated with meat and animal protein consumption. Importantly, one meta-analysis of 24 studies [148] concluded that soy consumption generally elicited either no weight change or weight gain. However, this same review concluded that isoflavone—and therefore genistein—consumption significantly reduced the body mass index (BMI) in postmenopausal women independent of soy protein intake.

### 3.2. Genistein and Breast Cancer

Breast cancer is the most common cancer in women in the United States, causing thousands of deaths each year. Given results from multiple literature reviews and studies [108,119,120,149,150], genistein has clearly shown a strong potential for breast cancer prevention. Women who eat more genistein, especially earlier in life, have a significantly decreased likelihood of developing breast cancer. These same women also have a lower risk of recurrence of treated breast cancer [119,120,121]. Genistein also inhibits the growth of human MCF-7 breast cancer cells at a concentration of 10^−5^ M [128], and even potentiates the anticarcinogenic effects of tamoxifen on the growth of ESR1-positive and HER2-overexpressing human breast cancer cells [149].

It is important to mention that genistein’s anticarcinogenic property is attenuated based on both the concentration of genistein and the cell surface receptors of the target cells. Although Wang et al. [128] demonstrated genistein’s ability to inhibit MCF-7 tumor growth at higher concentrations, at lower concentrations they saw stimulated growth. Furthermore, Pons et al. [151] illustrated that at concentrations representing genistein blood levels of individuals consuming a high-soy diet, genistein’s potentiating effects on breast cancer cells were influenced by the ESR1/ESR2 ratio of the cells. In this study, MCF-7 cells, which have a higher ESR1/ESR2 ratio, being treated with cisplatin, paclitaxel, or tamoxifen saw an increase in cell survivability when also treated with genistein. This suggests that genistein may have the potential to elicit counterproductive effects in women already being treated for a high ESR1/ESR2 ratio breast cancer using these common over-the-counter therapeutics [151]. Conversely, genistein was shown to have a harmless or beneficial effect when the procedure was repeated using cells with a low ESR1/ESR2 ratio (including both T47D cells and MCF-7 cells transformed to overexpress ESR2) [151]. Taken in context, this suggests that a genistein-supplemented regimen for treating breast cancer can be beneficial; however, it might be contraindicated for women whose tumors present with a high ESR1/ESR2 ratio.

Because of its numerous dose-dependent and receptor-influenced biological effects and its various metabolic pathways, it is difficult to conclude genistein’s role in breast cancer development and treatment. Though sufficient consumption has been shown to prevent breast cancer development [152], the literature is controversial regarding genistein’s effects on active cases of breast cancers. For example, a review published in 2000 focusing on genistein and breast cancer stated that the net result of genistein consumption on breast cancer activity or proliferation was inconclusive [119]. Furthermore, a meta-analysis of 164+ relevant studies in 2019 on genistein and breast cancer concluded that “the impact of dietary genistein intake on breast cancer remains unclear” [153]. Like the aforementioned studies, this review stated that variation in mode of intake, metabolism, menopausal status, estrogen receptor expression pattern, and gene mutations among individuals is key to determining the net effect of genistein consumption. These data suggest that further studies focused on the above factors and their interactions may yield more definitive answers and even future treatments for one of the most common cancers that affect women.

### 3.3. Genistein and Uterine Leiomyoma

Human uterine leiomyomas, also called fibroids, clinically affect about 40% of child-bearing aged women in the United States with symptoms of bleeding, dysregulation of the menstrual cycle, belly pain and infertility. Genistein’s effects on uterine leiomyomas is a rapidly expanding research subject, also appearing to follow a dose-dependent interaction pattern. Moore et al. [129] found that low in vitro concentrations of genistein (≤1 μg/mL) elicited proliferation in human uterine leiomyoma cells but did not do so in human uterine smooth muscle cells. However, higher exposure levels had inhibitory effects on both cell types causing cellular morphological changes, inhibiting cell proliferation, inducing apoptosis, and even causing targeted leiomyoma autophagy [154]. This type of biphasic dose-response is similar to what has been characterized regarding genistein and breast cancer [128]. It is also important to note that leiomyoma cells were more sensitive to the proliferative effects of genistein at a high dose (>1 μg/mL) than the smooth muscle cells, indicating a possible risk factor in terms of genistein consumption and fibroids.

There are many pathways by which genistein has been shown to affect leiomyoma growth; understanding these pathways is a critical step in revealing the mechanisms behind genistein-induced cell proliferation or inhibition and its therapeutic potential. Results from Di et al. [155] suggest that the inhibition described in Moore et al. [129] was the result of a high dose of genistein’s down-regulation of the TGF-β pathway, most notably activin A and Smad3. Although outlined in Eker rats, another pathway by which genistein was shown to inhibit leiomyoma cell proliferation was by acting as a ligand for peroxisome proliferator-activated receptor-γ [156]. Wang et al. [128] elaborated on these findings by hypothesizing that genistein’s emergent inhibitory effects at higher concentrations (>10^−5^ M) might occur through regulating the estrogen-responsive pS2 in contrast to its proposed activity at low concentrations.

Di et al. [157] demonstrated that low concentrations of genistein increased proliferation of uterine leiomyoma cells by rapidly associating with the IGF-1 receptor, causing interactions between ERα and IGF-IR, and activating the extracellular regulated kinase and MAP kinase pathways. Low concentrations of genistein have also been shown, in human leiomyoma cells, to activate MAPKp44/42, MSK1, and increase phosphorylation of histone H3 at serine10 (H3S10ph) [157]; these effects lead to increased cell proliferation, further demonstrating that genistein can even have epigenetic effects on human leiomyoma cells.

While there is sufficient evidence to conclude genistein can affect fibroids once formed, there is less evidence for associations between genistein consumption and the risk of fibroid incidence. As an example, Simon et al. [159] found that, across 328 women, there was no correlation between fibroid occurrence and urinary output of genistein (used as a proxy for blood genistein content). However, the inhibitory effects of genistein at high doses indicate that a requisite level of soy product consumption might be an important consideration in protecting patients with either predisposition towards or active fibroids.

### 3.4. Genistein and Endometriosis

Given genistein’s ability to mimic estrogen and endometriosis’ hormone-responsiveness [160], a reasonable hypothesis might assert that genistein could affect the incidence, severity, or timing of endometriosis that affects thousands of premenopausal women in the United States. Though 54 mg of oral genistein consumed daily has been shown to be an effective alternative treatment for managing endometrial hyperplasia in premenopausal women [161], much of the literature regarding its effects on endometriosis in women is conflicting. A large majority of it appears to be focused on in vivo rodent studies.

Regarding the incidence of endometriosis, it is uncertain as to whether genistein consumption affects a woman’s likelihood of developing the condition. One study encompassing over 500 American women found no significant correlation between endometriosis and urine concentration of genistein [162]. The same study further concluded that, among the women with moderate-to-severe endometriosis, there was no correlation between phytoestrogen consumption and disease severity. This study conflicts with results from another study which found that higher levels of urinary genistein were correlated with a reduction in advanced endometriosis risk [163]. However, this study is less generalizable because it only included women that were infertile and nulliparous. One might assume that comparing the prevalence of endometriosis across populations with large discrepancies in genistein consumption may help explain these conflicting results. However, women living in Asia and Japan, populations known to consume significantly more genistein than their Caucasian counterparts, develop endometriosis at a 1.5–3× greater rate than women in Western populations do [164]. Given that peer-reviewed studies have concluded that genistein may be associated with increased, decreased, or unchanged endometriosis risk, further controlled research is needed on a larger scale to explain any confounding variables, potential associations, or lack thereof.

The dispute over genistein’s anti-endometriosis effects appears to be less common across rodent studies. For example, Cotroneo and Lamartiniere [165] concluded that genistein’s effect on a rat model of endometriosis depended on the method of intake; subcutaneously injected genistein sustained intestinally implanted endometrial tissue, while dietary genistein did not. However, rats given oral genistein in Yavuz et al. [166] saw a significant regression of peritoneal endometriotic implants when compared to the control group. These two studies suggest genistein might have an inhibitory and even regressive effect on endometriotic cells, which is supported by studies showing that genistein inhibits expression of proinflammatory cytokines NF-κB, ESR2 [167], Bcl-2, COX-2, and PGE [168] in rodent models of endometriosis. There does not appear to be thorough research into why rodent studies are more conclusive in supporting genistein’s potential for treating endometriosis, but it may be due to several factors ranging from the paucity of human investigations, fundamental physiological differences between humans and rodents, confounding lifestyle variables, and more.

### 3.5. Genistein and Endometrial Cancer

In 2018, there were 89,929 related deaths and 382,069 new cases of endometrial cancer, globally [169]. The global incidence rate is also projected to pass 573,000 new cases by 2040 [170]. Genistein has been shown to inhibit endometrial cancer through a variety of direct and indirect pathways. For example, genistein was shown to suppress endometrial cancer cell proliferation in ECC-1 and RL-95-2 cell lines by decreasing expression of hTERT and ERα, leading to effects on both the AKT/mTOR and MAPK pathways [171]. Treatment with 5 mM of genistein was also sufficient to significantly affect Ishikawa cell proliferation and initiate downregulation of several prominent oncogenes, including the MAPK pathway-related genes (AA704613, MYC-associated zinc finger protein; and AA829383, mitogen-activated protein kinase), the cell cycle-related genes (AA789328, cyclin-dependent kinase (CDC2-like) 10; and W70051 M-phase phosphoprotein 9), and the cell migration and adhesion-related genes (AA283090 CD44 antigen; and N66616 phosphodiesterase 7A [172]. Further research confirmed a significant negative correlation among breast cancer survivors between consumption of genistein-containing herbal products and endometrial cancer incidence [173]. It appears that a significant body of research on the subject suggests that consumption of genistein has anti-endometrial cancer properties.

Understanding genistein’s effects on endometrial cancer-related hormones is critical in assessing genistein’s role in endometrial carcinogenesis. Though estrogen has been shown to induce the proliferation of endometrial cancer cells, genistein has been shown to suppress this process. Sampey et al. [174] showed that though genistein itself did not increase Ishikawa cell growth nor affect estrogen’s proliferative effects on an Ishikawa monoculture, 10–100 nM of genistein did suppress estrogen’s proliferative effects on a coculture including endometrial stromal cells along with Ishikawa cells. The same study further discussed how studies using ESR2-specific agonists yielded similar data. Since estrogen is a naturally occurring hormone in all women, this means that genistein is likely able, in vivo, to suppress endometrial cancer proliferation. This concept is further validated when considering results outlined in Zhang et al. [175] and Lee et al. [176], two literature analyses that discussed multiple epidemiological studies that showed a negative correlation between soya intake and endometrial cancer risk, and genistein and ovarian cancer risk, respectively.

### 3.6. Genistein and Polycystic Ovarian Syndrome

Given that polycystic ovarian syndrome (PCOS) is considered a possibly heritable disorder whose pathology might be partially hormonal in nature [177], there is a valid interest in genistein as a potential therapeutic for and effector of this disorder. Khani et al. [178] found that women with PCOS treated with a 3 month genistein regimen of 18 mg per 12 h saw decreases in circulating luteinizing hormone, serum triglyceride, LDL cholesterol, and testosterone; all of which are commonly increased in all PCOS patients. This evidence suggests that genistein can significantly improve the hormonal and lipid profile of women with PCOS, thereby reducing their likelihood of developing comorbid cardiovascular or metabolic disorders. This is supported by findings described in Jamilian and Asemi [179], a study that compared isoflavone supplementation to placebo in two groups of 35 women with PCOS. They found that the group that was supplemented daily with 50 mg of soy isoflavone for 12 weeks saw significant improvements in their hormonal and lipid profiles; the same group further showed decreased insulin resistance. Furthermore, another study utilizing a test diet including 35% soy protein found that women with PCOS that adhered to the diet saw improvements in BMI, glycemic control, circulating testosterone, and lipid profiles, alongside significant increases in circulating nitric oxide (NO) and glutathione (GSH) [180].

Using 36 mg/day, Romualdi et al. [181] reported similar findings with regards to cholesterol levels and triglycerides. However, this study was contradicted by both Khani et al. [178] and Jamilian and Asemi [179] in finding no significant changes in hormonal profiles and glycoinsulemic metabolism. However, this difference may be attributable to sample size (*n* = 12) or the profile of the sample population (all women in Romualdi et al. [181] had both hyperinsulemia and dyslipidemia alongside their PCOS).

Isoflavone supplementation has also been shown to clinically improve the gut health of women with PCOS. For example, after isoflavone intervention, a 50 mg isoflavones/day regimen over just three consecutive days improved predicted stool metagenomic pathways, microbial alpha diversity, and glucose homeostasis in PCOS patients. The effect was so profound that, post-treatment, these variables resembled the profile of the control group at baseline [182]. Though further testing is required, evidence suggests that dietary supplementation of genistein may be a viable natural option for treating many of the symptoms of PCOS [175,176,177,178,179].

### 3.7. Genistein and Cervical Cancer

Cervical cancer used to be the leading cause of cancer death of women in the United States; however, in the past 40 years, the number of cases of cervical cancer and cancer deaths have decreased significantly because of Pap tests and HPV vaccinations [183]. As with breast cancer, there exists conflicting evidence and controversial conclusions regarding genistein’s effects on the viability, incidence, and severity of cervical cancer. For example, while cervical cancer cells of the HeLa [184], CaSki and ME180 [185] lines have been shown to be sensitized to radiation therapy by high doses of genistein (20–40 mM), low concentrations of genistein (0.001–1 µM) have also been shown to promote HeLa cell proliferation and inhibit apoptosis via the PI3K/Akt-NF-κB pathway [133].

These results were directly challenged by Sahin et al. [186], who found that 25 μM genistein sensitized HeLa cells to cisplatin by inhibiting the NF-κB and Akt/mTOR pathways. The same results were also challenged by Hussain et al. [132], who found that genistein inhibited HeLa cell proliferation and promoted both apoptosis and cell cycle arrest at doses as low as 5 µM, becoming more effective with higher doses tested up to 150 µM.

The difference between the conclusions of these studies seems to lie in the concentration of genistein used to expose HeLa cervical cancer cells. Similar to the biphasic concentration-dependent responses observed in uterine leiomyoma [129] and MCF-7 breast cancer cells [128] (noted in Figure 3), HeLa cells appeared to show the same nonmonotonic response to genistein across these three studies; exhibiting proliferation at lower concentrations and suppression at higher concentrations. Most notably, increasing the dosage meant that genistein had the opposite effect on the NF-κB pathway. Though more research is needed—especially in terms of epidemiological and human in vivo evidence—to come to a sound conclusion regarding genistein and cervical cancer, this evidence suggests that using genistein at controlled high concentrations could be an effective treatment for both inhibiting the growth of and radiosensitizing cervical cancer cells for therapy.

### 3.8. Genistein and Menopause (Hormone Regulation)

Menopause is marked by a series of physiological changes linked to a reduction in bodily estrogen and progesterone production, potentially eliciting symptoms such as hot flashes secondary to vasomotor dysfunction, sweating, thinning of vaginal membranes, mood effects, sleep insufficiency, and more [187]. It occurs in women between ages 40–58, and even older, yet the age of onset can be affected by multiple factors including smoking, contraceptive use, BMI, and more [188]. It is unlikely that genistein influences the timing of menopause onset itself; despite a large disparity in genistein consumption between Asian and Caucasian women, they both typically experience menopause at around the same age [189,190].

Genistein has been thoroughly explored for its potential use in postmenopausal hormonal replacement therapy in alleviating the severity of menopausal symptoms. This is largely because although current hormone-replacement therapies are available, many of them may increase the risk of thromboembolism, cancer, stroke, and other complications [191]. The use of genistein as a form of hormone replacement therapy is common [114], and has been shown to be significantly more effective than a placebo at combatting several of the post-menopausal symptoms and physiological effects in women.

Double-blind studies showed that 54 mg of daily oral genistein reduced hot flashes without negatively affecting endometrial thickness, liver function, or blood physiology in postmenopausal women [192]. The same dosage has also been shown to reduce bone resorption while increasing bone deposition [193], improve brachial arterial vasodilation and perfusion capability [194], enhance endothelium function as effectively as estrogen/progesterone treatment [195], and even showed cardioprotective activity in postmenopausal women [196].

Even considering different populations, those that consume more genistein such as Asian women have a significantly decreased propensity towards postmenopausal hot flashes [114]. Reinforcing this notion, a 2016 meta-analysis of 62 clinical trials across 6653 postmenopausal women found that phytoestrogen supplementation was significantly correlated with reductions in the number of daily hot flashes and general vaginal dryness [197]. The literature appears to mostly support genistein supplementation as a viable option for reducing many of the symptoms of menopause (Table 3).

## 4. Clinical Therapeutic Options

Because of its numerous positive biological effects, genistein has begun to see widespread medicinal use. Over the counter genistein supplements are marketed to the general public using buzzwords such as “life extension, wellbeing, health supplement,” and more [202]; however, even “Amazon’s Choice” genistein supplement lacks citations, instead stating that these claims have not been validated by the FDA in the fine print [203]. While there is a large body of evidence suggesting the numerous health benefits of genistein for nearly all populations, it is important that the public also be informed of the risks associated with supplementation. This is especially relevant and concerning considering many of these marketed supplements are individual pills containing 125+ mg pure genistein aglycone each [202], greater than 5× the total daily average whole genistin and genistein consumption values for Chinese [62] and Japanese adults [61]. Values in this range are within the realm of doses given in genistein’s clinical trials [109]. Despite the therapeutic potential of these significantly larger dosages, they also carry increased risks [116,117,118]. Given the need for further investigation into maximizing the benefits and minimizing the potential side effects, genistein supplementation is currently not recommended without first consulting a pharmacist or physician [204]. Across much of the literature, the most consistent and safe results appear to be found at genistein consumption levels similar to those found in traditional Asian diets [108,110,112,120,121,193,205].

As previously mentioned, genistein has been used in human clinical trials for purposes such as restricting growth and growth factor activity in cancer cells [109]; a 2008 literature review [149] reported that across 20+ studies, genistein has been shown to be a strong potentiator of antitumor chemotherapeutics, including tamoxifen [150]. Genistein was even found to increase the sensitivity of renal [198], prostate [206], esophageal [199], and cervical cancers [185] to radiation therapy. Genistein therapies show great potential; they utilize genistein’s dual effects that are cell- and organ-specific, hormone receptor content-mediated, and concentration-dependent to improve the clinical outlook of a broad range of women’s diseases.

## 5. Conclusions

The geographic distribution of soya product consumption has resulted in differential serum concentrations of genistein globally. We have evaluated and summarized research evidence from current in vivo and in vitro studies, clinical observations, and epidemiological surveys to show that genistein has been reported to have dual effects in women’s health when all data are taken under consideration. The effects of genistein appear to be dose-dependent and varies by individuals and suggests that genistein’s effects may be dependent on the levels of consumption, serum concentrations, and other factors that may contribute to its beneficial effects in women’s health, disease prevention, and treatment. However, there have been inconclusive beneficial effects of genistein reported in women and in in vivo animal studies; conversely, even adverse effects have been observed at lower concentrations in in vitro and in in vivo animal models. Therefore, the duplicity of genistein in women’s health is that it has been reported to serve as a possible beneficial or therapeutic agent in some instances and as an endocrine disruptor in other situations.

## Figures and Tables

**Table 1:** Genistein content in consumable products.

Food	Mean Genistein Concentration ^a^ (mg Genistein/ 100 g Food)	Standard Deviation	References
Textured Soy Flour	89.42	26.96	[7,8,9,10,11,12,13,14,15]
Instant Beverage Soy Powder	62.18	3.69	[14,16,17,18,19,20]
Soy Protein Isolate	57.28	14.17	[7,14,16,19,21,22,23,24,25,26,27,28,29]
Meatless Bacon Bits	45.77	0.11	[13]
Kellog’s Smart-Start Soy Protein Cereal	41.90	N/A ^b^ (*n* < 3)	[13]
Natto	37.66	7.85	[30,31,32,33,34,35,36]
Uncooked Tempeh	36.15	17.64	[11,14,16,29,31,37,38,39]
Miso	23.24	8.37	[14,16,17,30,31,33,35,36,40,41,42,43]
Sprouted Raw Soybeans	18.77	11.22	[23,32,40,44,45,46,47,48,49]
Cooked Firm Tofu	10.83	3.98	[30,40,50]
Red Clovers	10.00	0.00	[51]
Worthington FriChik canned meatless chicken nuggets (prepared)	9.35	N/A (*n* < 3)	[31]
American Soy Cheese	8.70	N/A (*n* < 3)	[30]
Kellog’s Kashi Go-Lean Cereal	7.70	N/A (*n* < 3)	[13]
Chocolate Power Bar	3.27	N/A (*n* < 3)	[44]
Hoisin Sauce	3.25	N/A (*n* < 3)	[13]
Cake-Type Plain Doughnuts	2.44	1.11	[13,40]
Raw Pistachios	1.75	N/A (*n* < 3)	[40,52]
Reconstituted Infant Formula (Abbot Nutrition)	1.37	0.37	[53,54]
Cooked USDA Commodity Beef Patties	1.09	0.42	[31]
Fat Free Frankfurter Beef	1.00	N/A (*n* < 3)	[13]
Raw Chicken Breast Tenders	0.25	N/A (*n* < 3)	[13]
Raw White Grapefruit	0.03	N/A (*n* < 3)	[44]
Whole Raw Eggs	0.02	N/A (*n* < 3)	[44,45]
Mature Raw Black Beans	0.00	0.00	[44,55,56]

^a^ Data summarized from [4] ^b^ Not Applicable.

**Table 2:** Genistein levels in populations worldwide.

Population	Number of Subjects	Sample Type	Quantified Genistein	References	Year
Healthy infants in Pennsylvania, collected at the Children’s Hospital of Philadelphia and its affiliated clinics		Blood, urine, and saliva samples from cow- and breast-milk-fed infants	Large majority, except for cow’s milk-formula-fed infants, below LOD (<27 ng/mL in blood, <1.4 ng/mL in saliva, and <0.8 ng/mL in urine)	[57]	2009
	165	Urine (cow-formula-fed infants)	13.6 ng/mL		
		Blood (soy-formula-fed infants)	890.7 ng/mL (median)		
		Urine (soy-formula-fed infants)	7220 ng/mL (median)		
		Saliva (soy-formula-fed infants)	10.9 ng/mL (median)		
Cohort of women in Philadelphia, PA, USA	451	Daily consumption	2.4–3.9 mg (average)	[58]	2008
Subgroup of larger cohort of women in Philadelphia, PA, USA	27	Daily urine excretion	136.4 ng genistein/mg creatine (average)		
Adult participants from Ireland, Italy, the Netherlands, and the UK		Daily consumption (Ireland)	0.368 mg/day (average)	[59]	2003
	7312	Daily consumption (Italy)	0.302 mg/day (average)		
		Daily consumption (the Netherlands)	0.516 mg/day (average)		
		Daily consumption (the UK)	0.389 mg/day (average)		
Women of various racial and ethnic groups across the US	1550	Daily consumption (White women)	3.6 μg genistein/day (average)	[60]	2006
	935	Daily consumption (African American women)	1.7 μg genistein/day (Average)		
	286	Daily consumption (Hispanic women)	0 μg genistein/day (average)		
	185	Daily consumption (Chinese women)	3534 μg genistein/day (average)		
	195	Daily consumption (Japanese women)	6788 μg genistein/day (average)		
Adults from various regions of Japan	215	Daily consumption	14.5–18.3 mg genistein/day	[61]	2001
		Serum level	475.3 nmol genistein/liter of serum		
		Daily excretion in urine	14.2 μmol genistein/day		
Chinese men	48	Daily consumption	19.4 ± 12.36 mg/day	[62]	2007
Adult (20–39 years old) women from the UK	20	Plasma genistein concentration of women that rarely consumed soy products	14.3 nmol/L (geometric mean)	[63]	2001
	20	Plasma genistein concentration of women that drank no soy milk but ate some solid soya foods	16.5 nmol/L (Geometric mean)		
	20	Plasma genistein concentration of women that drank 0.25 pints of soy milk daily and ate some solid soya foods	119 nmol/L (geometric mean)		
	20	Plasma genistein concentration of women that drank 0.5+ pints of soy milk daily and ate solid soya foods regularly	378 nmol/L (geometric mean)		

**Table 3:** Summary of 62 human studies and literature reviews of human studies on genistein’s effects on women’s health—both in vivo and in vitro studies are included.

Category of Studies’ Conclusions Regarding Genistein/Whole Isoflavones/Genistein Metabolites	Total Number of Studies (Number Included in the Exposure Testing Range/Daily Dosage Column In Vivo) ^a^	Exposure Testing Range In Vitro (µM)	Daily Dosage Testing Range In Vivo (mg)	References
Evidence suggests effects are primarily beneficial	42 (27)	Genistein: 2.0–370	Genistein: 36–600 (all doses above 54 were in one study) Genistein Mode: 54 (7 studies) Whole Isoflavones: 40–165 Soy Intakes/Week: 0.76–12.0	[67,68,95,108,109,110,111,112,120,121,125,132,143,144,145,146,147,149,150,152,161,171,172,173,175,176,178,179,180,182,184,185,186,192,193,194,195,196,197,198,199,200]
Evidence suggests effects are debated/inconclusive, but does suggest potential benefits	11 (9)	Genistein: 0.0037–185	Genistein: 30–54 Whole Isoflavones: 45	[108,114,119,128,129,151,154,155,163,174,181]
Evidence suggests effects are debated/inconclusive, and does not show any potential for benefits	4 (2)	Genistein: 1–10	Whole Isoflavones: 33.3–300	[148,153,159,162]
Evidence suggests effects are primarily detrimental	5 (5)	Genistein: 0.001–3.7	N/A ^b^	[127,133,157,158,201]

^a^ Omitted studies include those which reported measures that could not be converted to fit this table. ^b^ Not applicable.

## Data Availability

Not Applicable.
